# The Prognostic Value of Bone Morphogenetic Proteins and Their Receptors in Lung Adenocarcinoma

**DOI:** 10.3389/fonc.2021.608239

**Published:** 2021-10-22

**Authors:** Wangyang Meng, Han Xiao, Rong Zhao, Dong Li, Kuo Li, Yunchong Meng, Jiaping Chen, Yangwei Wang, Yongde Liao

**Affiliations:** ^1^ Department of Thoracic Surgery, Union Hospital, Tongji Medical College, Huazhong University of Science and Technology, Wuhan, China; ^2^ Department of Dermatology and Sexology, Tongji Hospital, Tongji Medical College, Huazhong University of Science and Technology, Wuhan, China

**Keywords:** lung adenocarcinoma, prognosis, risk model, bone morphogenetic proteins, bone morphogenetic protein receptors

## Abstract

**Background:**

Bone morphogenetic proteins (BMPs) regulate tumor progression *via* binding to their receptors (BMPRs). However, the expression and clinical significance of BMPs/BMPRs in lung adenocarcinoma remain unclear due to a lack of systematic studies.

**Methods:**

This study screened differentially expressed BMPs/BMPRs (deBMPs/BMPRs) in a training dataset combining TCGA-LUAD and GTEx-LUNG and verified them in four GEO datasets. Their prognostic value was evaluated *via* univariate and multivariate Cox regression analyses. LASSO was performed to construct an initial risk model. Subsequently, after weighted gene co-expression network analysis (WGCNA), differential expression analysis, and univariate Cox regression analysis, hub genes co-expressed with differentially expressed BMPs/BMPRs were filtered out to improve the risk model and explore potential mechanisms. The improved risk model was re-established *via* LASSO combining hub genes with differentially expressed BMPs/BMPRs as the core. In the testing cohort including 93 lung adenocarcinoma patients, immunohistochemistry (IHC) was performed to verify BMP5 protein expression and its association with prognosis.

**Results:**

BMP2, BMP5, BMP6, GDF10, and ACVRL1 were verified as downregulated in lung adenocarcinoma. Survival analysis identified BMP5 as an independent protective prognostic factor. We also found that BMP5 was significantly correlated with EGFR expression and mutations, suggesting that BMP5 may play a role in targeted therapy. The initial risk model containing only BMP5 showed a significant correlation (HR: 1.71, 95% CI: 1.28−2.28, *p*: 3e-04) but low prognostic accuracy (AUC of 1-year survival: 0.6, 3-year survival: 0.6, 5-year survival: 0.63). Seventy-nine hub genes co-expressed with BMP5 were identified, and their functions were enriched in cell migration and tumor metastasis. The re-established risk model showed greater prognostic correlation (HR: 2.58, 95% CI: 1.92–3.46, *p*: 0) and value (AUC of 1-year survival: 0.72, 3-year survival: 0.69, and 5-year survival: 0.68). IHC results revealed that BMP5 protein was also downregulated in lung adenocarcinoma and higher expression was markedly associated with better prognosis (HR: 0.44, 95% CI: 0.23–0.85, *p*: 0.0145).

**Conclusion:**

BMP5 is a potential crucial target for lung adenocarcinoma treatment based on significant differential expression and superior prognostic value.

## Introduction

Bone morphogenetic proteins (BMPs) were initially identified as factors that induce ectopic bone formation (1). Since the first isolation of BMP activity from extracts of bovine bone in 1988 (2), more than a dozen BMPs have been identified (3). Except for the identification of BMP1 as a metalloproteinase (MMP), all other BMPs were found to be novel members of the transforming growth factor β (TGF-β) superfamily (4). Like other TGF-βs, BMPs could regulate skeletal biology and embryonic development through two types of serine-threonine kinase transmembrane receptors, type I (ACVRL1, ACVR1, BMPR1A, and BMPR1B) and type II (BMPR2, ACVR2A, and ACVR2B) (5). Although bone-inducing activity is unique to BMPs among TGF-βs (6), accumulating evidence has found that BMPs contribute to the process of tumorigenesis and regulate cancer progression through various stages (5, 7).

BMPs binding to the tetramer complex assembled from homodimeric type I/type II receptors lead to activation of the type I receptors *via* the phosphorylation of the type II receptor and subsequent phosphorylation of Smad family and other signaling proteins (7). Dysregulated BMP/BMPR signaling perturbs the dynamic equilibrium of cytoplasmic constituents and the ordered transcription of several genes, increasing the risk of the development of diverse cancers (8). The spectrum of cancers where BMPs/BMPRs have been reported to play a role is large. For example, BMP2 promotes invasion and bone metastasis of breast cancer through the Smad pathway (9), and prostate cancer cells metastasize while undergoing epithelial–mesenchymal transition (EMT) in response to BMP7 (10). However, there is complexity and contradiction in the research focused on the function of BMPs. A recent study found that activation of BMP4-SMAD7 suppressed breast cancer metastasis (11), where another study supported BMP4 as a promoter of prostate tumor growth in bone through osteogenesis (12). The controversy has driven further research of BMPs/BMPRs in cancers, focused on tumorigenesis, proliferation, invasion and metastasis, angiogenesis, and lymph node metastasis (13, 14).

A greater number of people die from lung cancer than prostate, colon, breast, and kidney cancers combined. This is partially due to a lack of precise diagnosis in the early stages and effective treatment options in the advance stages (15). Studies have reported that BMP2/BMP4 regulates lung development and that the BMP signaling cascade is reactivated to promote lung tumorigenesis (16, 17). JL5, an inhibitor target for BMPs, has been confirmed to suppress tumor cell survival signaling and induce regression of human lung cancer (18). BMPs/BMPRs may be the novel biomarkers for lung cancer diagnosis as well as effective therapeutic targets. However, only these researches are far from enough compared with the above other cancers. To date, research has still not determined which BMPs are essential, the roles of BMPs, and their mechanisms in lung cancer.

**Figure 1:**
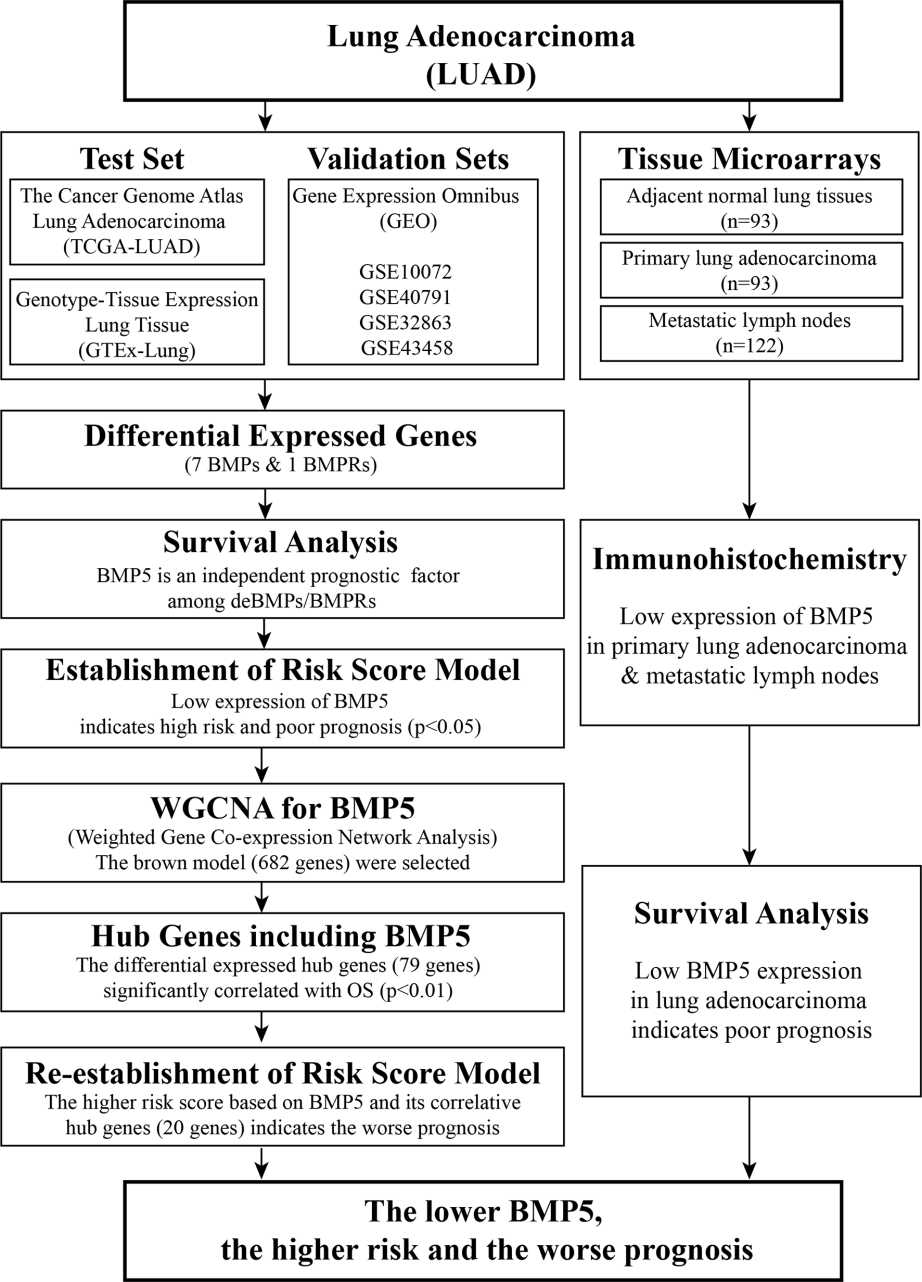
Workflow of identification of differentially expressed bone morphogenetic proteins (BMPs)/bone morphogenetic protein receptors (BMPRs) and evaluation of their prognostic value in lung adenocarcinoma.

As shown above, the BMP family is large, and its signaling in cancer, including lung cancer, is complex. At present, there is still a lack of systematic description of BMP/BMPR expression levels in lung cancer tissue, especially lung adenocarcinoma. The current study analyzed the expression profiles, evaluated the prognostic value, and preliminarily analyzed the potential roles of BMPs/BMPRs in lung adenocarcinoma (**Figure 1**). These results will be helpful for the follow-up exploration of the potential use of BMPs/BMPRs in the diagnosis and treatment of lung adenocarcinoma.

## Materials and Methods

### Gene Expression Profile Data

The gene expression profile data in the current study combined the RNA-seq transcriptome data of lung adenocarcinoma (LUAD) cohort from The Cancer Genome Atlas (TCGA) and lung tissue from the Genotype-Tissue Expression (GTEx) from the University of California Santa Cruz (UCSC) Xena platform (https://xenabrowser.net/datapages/). The normalized gene expression level of the TCGA-LUAD cohort was utilized to evaluate the expression of BMPs/BMPRs in lung adenocarcinoma tissues (*n* = 526) compared with normal lung tissues (*n* = 59). The GTEx (https://commonfund.nih.gov/GTEx/) project provided us the RNA-seq data of normal lung tissue (GTEx-LUNG, *n* = 288), which effectively reduced the bias resulting from the large gap of sample sizes between lung adenocarcinoma and normal lung tissues in TCGA-LUAD (19).

### Selection of BMPs and Their Receptors (BMPRs)

The BMP signaling pathway is an intricate system with more than 20 ligands. Because BMP1 is the only MMP, it was excluded from our study. Due to multiple approaches used for the identification of BMPs, some were described with different names such as growth and differentiation factors (GDFs) and osteogenic proteins (OPs). To avoid confusion, only the terms “BMP” and “GDF” are used in this study. As for BMPRs, there are four type I receptors and three type II receptors. We extracted the expression matrix of 11 BMPs and 7 BMPRs and the clinical information of the samples for subsequent bioinformatics analysis.

### Screening of Differentially Expressed Genes

To screen the differentially expressed genes among 11 BMPs and 7 BMPRs in lung adenocarcinoma, the package “Limma” in R/Bioconductor software was applied to analyze the expression of 18 genes in 526 tumor tissues and 347 normal lung tissues. The results of the differentially expressed gene (DEG) analysis are presented in the form of a heatmap, and the violin plot was used to visualize the overall expression levels of BMPs/BMPRs in lung adenocarcinoma and normal lung tissues. We defined the genes with logFC value (the logarithm of fold change) >1 or ≤1 and *p <*0.05 as the DEGs.

### Validation of Gene Expression Omnibus Datasets

Although the amount of data combining TCGA-LUAD and GTEx-LUNG was large, the DEG analysis between no paired cancer and normal tissues could be affected by individual differences. To further validate the ubiquity of differential expression of BMPs/BMPRs between lung adenocarcinoma and normal lung tissues, four gene expression profile datasets [GSE10072 (20), GSE40791 (21), GSE32863 (22), and GSE43458 (23)] of lung adenocarcinoma were obtained from the Gene Expression Omnibus (GEO) and met the following criteria: 1) RNA-seq transcriptome data were obtained from tissues of patients with lung adenocarcinoma, 2) lung adenocarcinoma tissues were paired with adjacent normal lung tissues, 3) all samples were collected with complete clinicopathologic features, 4) sample size must be more than 50, and 5) the relevant results of the data must be published in a high-quality journal.

### Survival Analysis of Differentially Expressed BMPs/BMPRs

After removing 347 normal tissues, we analyzed the prognostic value of the differentially expressed BMPs/BMPRs screened in 526 lung adenocarcinoma tissues. The “corrplot” package was used for visualization of correlation among the BMPs/BMPRs, and the “survminer” package was used to draw the Kaplan–Meier curve. The Kaplan–Meier plotter (http://kmplot.com/), an online database capable of assessing the association of genes on survival in four types of cancer (lung, breast, gastric, and ovarian cancer), was also applied to verify the prognostic value of BMPs/BMPRs in lung adenocarcinoma patients (*n* = 719) (24). Next, the forest plot was used to present the results of the Cox multivariate regression analysis evaluating the association between differentially expressed BMPs/BMPRs and overall survival (OS).

### Construction of the Risk Model

To predict the clinical outcomes of lung adenocarcinoma patients using BMPs/BMPRs, the R package “glmnet” was utilized to construct the risk model using the least absolute shrinkage and selection operator (LASSO) Cox regression algorithm in the TCGA-LUAD dataset. Based on the minimum criteria, part of the differentially expressed BMPs/BMPRs was selected out and the risk scores were calculated from the coefficients of the risk model. Then, the TCGA-LUAD dataset was separated into low- and high-risk groups based on the mean risk score. The prognostic value of the risk model was evaluated through Kaplan–Meier survival analysis, ROC curve, and univariate and multivariate Cox regression analyses.

### Weighted Gene Correlation Network Analysis

To explore the potential mechanism of BMPs/BMPRs involved in the risk model, the weighted gene correlation network was constructed using the weighted gene correlation network analysis (“WGCNA”) package in R (25). In TCGA-LUAD, the top 5,000 genes were selected in decreasing order of median absolute deviation (MAD), and their clustering modules were identified based on clinicopathologic features (including the expression of BMPs/BMPRs selected in the above model) (26). The correlation between module eigengenes and clinicopathologic features was calculated for the identification of the highly relevant modules.

### Identification of Hub Genes and Re-Establishment of the Risk Model

We selected the genes in the module that were highly relevant to the expression of BMPs/BMPRs in the risk model. Next, the “ClusterProfiler” R package was utilized for Gene Ontology (GO) enrichment analysis of the module genes (27). Then, DEG analysis and Cox multivariate regression analysis were carried out to further screen the hub genes with differential expression and significant prognostic value (*p* < 0.001) from the module genes. Finally, the hub gene prognostic value was weighted *via* LASSO regression, and the risk model was re-established.

### Patients and Tissue Samples

A total of 93 lung adenocarcinoma patients who received surgical resection at Tongji Hospital of Huazhong University of Science and Technology, Tongji Medical College (Wuhan, China) and who accepted medical follow-up that continued until July 2021 were included. Paraffin specimens from these patients were collected at Tongji Hospital between 2014 and 2018. All 93 patients were histologically diagnosed with primary lung adenocarcinoma. The study was approved by the Institutional Ethics Committee of Tongji Medical College, Huazhong University of Science and Technology, and written informed consent was obtained from all of the patients before surgery.

### Immunohistochemistry Staining

Immunohistochemistry (IHC) staining was performed for the tissue microarrays (TMA) which contain 93 paired paraffin-embedded lung adenocarcinoma tissues and adjacent normal tissues together with 122 metastatic lymph nodes. The IHC kit (Gene Tech Co. Ltd., Shanghai, China) was used according to the instructions of the manufacturer. Primary antibody of rabbit anti-BMP5 antibody (dilution 1:200) was purchased from Cusabio Co. Ltd. (Wuhan, China). We evaluated BMP5 expression levels in each specimen with a semiquantitative immunoreactivity scoring system, which ranged from 1 to 8 and was equal to the sum of the intensity of IHC staining (1, negative; 2, weakly positive; 3, moderately positive; and 4, strongly positive) and the percentage of positive cells (1, ≤25% positive cells; 2, 25%–50% positive cells; 3, 50%–75% positive cells; and 4, >75% positive cells). The mean IHC score was chosen as the cutoff value to define low and high BMP5 expression. Protein expression levels of BMP5 were independently scored by two pathologists with the blind method.

### Statistical Analysis

Normally distributed data were analyzed using Student’s *t*-test, and non-normally distributed data were analyzed using Mann–Whitney tests, after a normality check. The Pearson test was employed to evaluate the correlation among BMPs/BMPRs, and Kendall rank correlation coefficient tests were used to check the correlation of these DEGs with clinicopathologic features. The prognostic value of BMPs/BMPRs was evaluated using Kaplan–Meier survival analysis and Cox multivariate regression analysis. Differences were considered to be statistically significant at *p <*0.05.

## Results

### The Differential Expression of BMPs/BMPRs in Lung Adenocarcinoma

**Figure 2:**
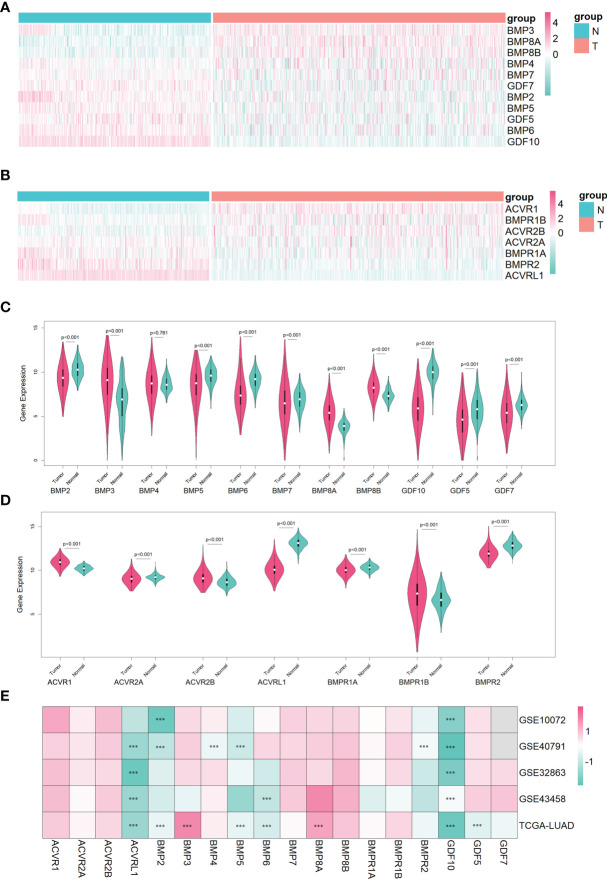
The identification of differentially expressed BMPs/BMPRs in the training dataset combining TCGA-LUAD and GTEx-LUNG, and the verification of four GEO datasets in lung adenocarcinoma. **(A)** Heatmap of differential expression analysis for 11 BMPs in the training dataset. **(B)** Heatmap of differential expression analysis for seven BMPRs in the training dataset. **(C)** Violin plot indicating the expression of 11 BMP expression between tumor and normal tissues in the training dataset. **(D)** Violin plot indicating the expression of seven BMPRs between tumor and normal tissues in the training dataset. **(E)** Heatmap of differential expression analysis for 11 BMPs and 7 BMPRs in four GEO validation datasets. **(C, D)** The white point represents the median Q2, the black bar ranging from the lower quartile Q1 to the upper quartile Q3 represents the dispersion and symmetry of the non-abnormal data, the black line running through the violin plot represents the maximum and minimum non-abnormal values, and the outer shape of the violin plot is the kernel density estimation. **(E)** Every cell representing differentially expressed BMPs/BMPRs in each dataset is labeled with *p*-value (***<0.001).

In the lung adenocarcinoma gene expression profile dataset, 526 cases were tumor tissues (all from TCGA-LUAD) and 347 cases were normal tissues (288 from GTEx-LUNG and 59 from TCGA-LUAD). To explore the differential expression of BMPs/BMPRs, we extracted and compared the expression levels of 18 BMPs/BMPRs between tumor and normal tissues in the above datasets (**Supplementary Table S1**). Among 11 BMPs (**Table 1**), BMP3 and BMP8A expression levels were upregulated, and 5 BMPs (BMP2, BMP5, GDF5, BMP6, and GDF10) downregulated in tumor tissues compared with normal tissues (**Figure 2A**). For seven BMPRs (**Table 2**), only ACVRL1 was significantly downregulated in tumor tissues, and significant differential expression of other BMPRs was not observed compared with normal tissues (**Figure 2B**). The violin plot describing the distribution of BMP expression in tumor and normal tissues suggested that GDF10 was the most significantly downregulated BMP (**Figure 2C**), and the downregulating degree of ACVRL1 was obvious from the violin plot of BMPRs (**Figure 2D**).

**Table 1:** The differential expression of 11 BMPs in lung adenocarcinoma.

BMP	logFC	AveExpr	*t*	P.Value	adj.P.Val	*B*	Change
BMP3	2.2347	7.9865	13.9163	6.28E-40	1.73E-39	79.2211	Up
BMP8A	1.6218	4.7445	20.7607	3.38E-78	1.86E-77	167.0745	Up
BMP8B	0.8561	7.8675	14.1697	3.39E-41	1.24E-40	82.1275	Stable
BMP4	−0.0274	8.6173	−0.2980	0.7658	0.7658	−8.2793	Stable
BMP7	−0.3422	6.6755	−2.6837	0.0074	0.0082	−4.7352	Stable
GDF7/BMP12	−0.9230	5.7625	−9.9232	4.55E-22	5.56E-22	38.3649	Stable
BMP2	−1.0554	9.7086	−11.7204	1.41E-29	2.58E-29	55.5216	Down
BMP5	−1.1252	8.8726	−10.2124	3.26E-23	5.12E-23	40.9767	Down
GDF5/BMP14	−1.2756	5.0990	−10.0676	1.23E-22	1.69E-22	39.6619	Down
BMP6	−1.4841	8.2127	−13.4882	8.09E-38	1.78E-37	74.3865	Down
GDF10/BMP3B	−4.0522	7.4526	−34.8951	4.87E-168	5.35E-167	373.6674	Down

**Table 2:** The differential expression of seven BMPRs in lung adenocarcinoma.

BMPR	logFC	AveExpr	*t*	P.Value	adj.P.Val	*B*	Change
ACVR1/ALK2	0.7063	10.6461	22.8549	5.45E-91	1.91E-90	195.8391	Stable
BMPR1B	0.4875	6.9399	4.2376	2.50E-05	2.50E-05	−0.1824	Stable
ACVR2B/ACTR2B	0.4484	8.9466	9.6674	4.51E-21	7.89E-21	35.3598	Stable
ACVR2A/ACTR2	−0.1720	9.1097	−4.9335	9.67E-07	1.13E-06	2.9367	Stable
BMPR1A	−0.2687	10.1461	−8.5346	6.18E-17	8.65E-17	25.9365	Stable
BMPR2	−0.9305	12.2931	−22.3267	1.03E-87	2.41E-87	188.3003	Stable
ACVRL1/ALK1	−2.9977	11.3011	−59.7668	7.55e-311	5.28e-310	702.0866	Down

To verify the differential expression of BMPs/BMPRs in the above dataset, four GEO datasets of lung adenocarcinoma meeting the criteria were selected (**Table 3**). The logFC values were obtained from differential expression analysis of four GEO datasets. The positive of logFC indicated upregulation of genes and the negative indicated downregulation in tumor tissues compared with normal tissues. The heatmap drawn from logFC and *p*-values showed that the four downregulated BMPs (BMP2, BMP5, BMP6, and GDF10) and ACVRL1 were verified among four GEO datasets, and the trend of GDF10 downregulation was the most stable (**Figure 2E**).

**Table 3:** Characteristics of four GEO datasets in lung adenocarcinoma.

GSE	GPL	Tissue	Sample Type		Reference	PMID
			Normal	Tumor		
GSE10072	GPL96	LUAD	49	58	Landi, Dracheva et al. (20)	18297132
GSE40791	GPL570	LUAD	100	94	Zhang, Foreman et al. (21)	23187126
GSE32863	GPL6884	LUAD	58	58	Selamat, Chung et al. (22)	22613842
GSE43458	GPL6244	LUAD	30	80	Kabbout, Garcia et al. (23)	23659968

LUAD, lung adenocarcinoma.

### Survival Analysis of Differentially Expressed BMPs/BMPRs

**Figure 3:**
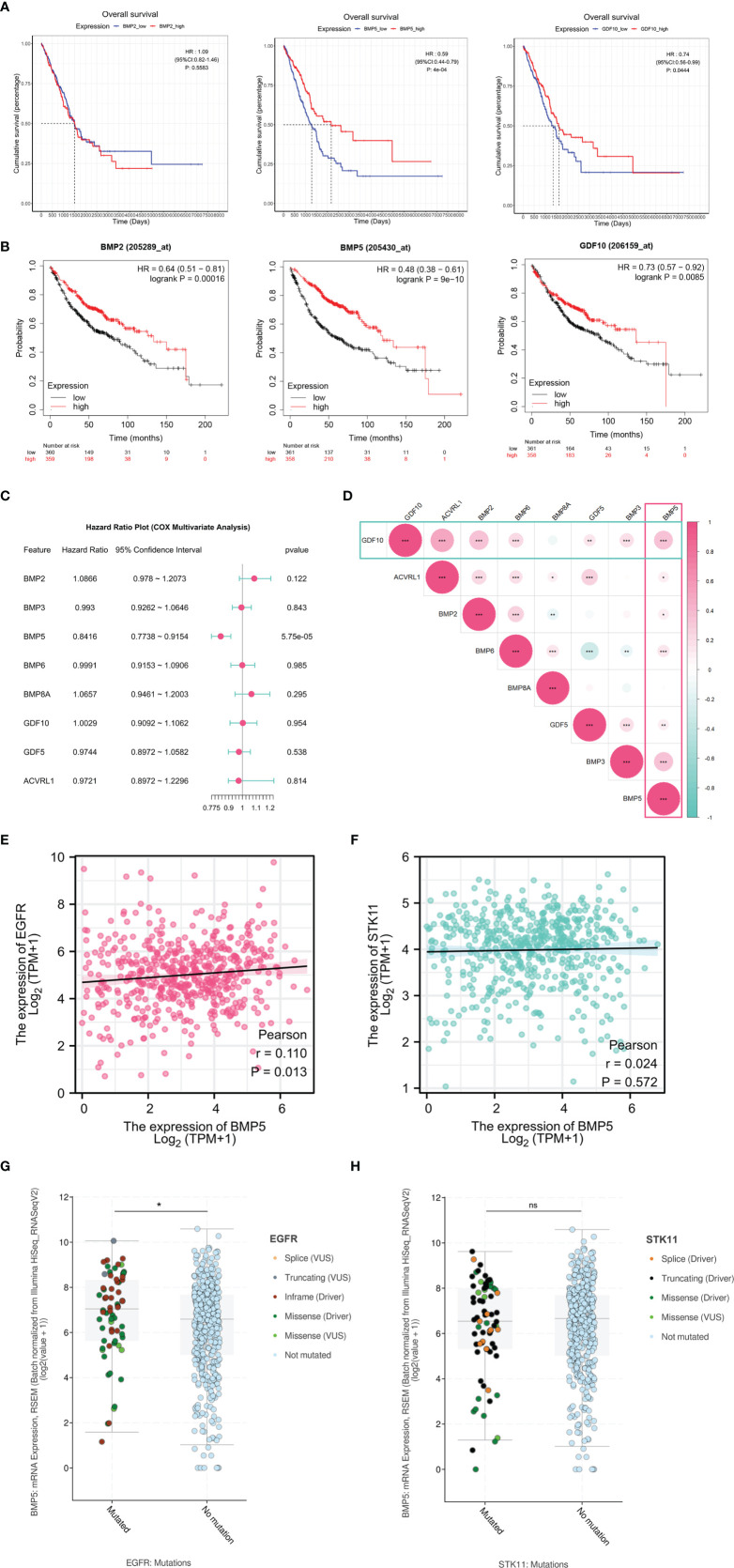
The survival analysis of differentially expressed BMPs/BMPRs. **(A)** The Kaplan–Meier curve of BMP2, BMP5, and GDF10 in the tumor samples of TCGA-LUAD (*n* = 513). BMP2 (HR: 1.09, 95% CI: 0.82–1.46, *p*: 0.5583), BMP5 (HR: 0.59, 95% CI: 0.44–0.79, *p*: 4e-04 < 0.001), and GDF10 (HR: 0.74, 95% CI: 0.56–0.99, *p*: 0.0444 < 0.05). **(B)** The Kaplan–Meier curve of BMP2, BMP5, and GDF10 in lung adenocarcinoma of Kaplan–Meier plotter (*n* = 719). BMP2 (HR: 0.64, 95% CI: 0.51–0.81, *p*: 0.00016 < 0.001), BMP5 (HR: 0.48, 95% CI: 0.38–0.61, *p*: 9e-10 < 0.001), and GDF10 (HR: 0.73, 95% CI: 0.57–0.92, *p*: 0.0085 < 0.001). **(C)** Forest plot of the multivariate Cox proportional hazard model including eight differentially expressed BMPs/BMPRs, BMP5 (HR: 0.8416, 95% CI: 0.7738–0.9154, *p*: 5.75e-05 < 0.001). **(D)** The correlation among eight differentially expressed BMPs/BMPRs with coefficients in the form of circle size and *p*-value (*<0.05, **<0.01, ***<0.001). **(E)** Scatter plot of correlation between the mRNA expression levels of BMP5 and EGFR (*p* = 0.013, *r* = 0.110). **(F)** Scatter plot of correlation between the mRNA expression levels of BMP5 and STK11 (*p* = 0.572, *r* = 0.024). **(G)** Boxplot of correlation between EGFR mutations and BMP5 mRNA expression (*p* = 0.0372, *<0.05, **<0.01, ***<0.001). **(H)** Boxplot of correlation between STK11 mutations and BMP5 mRNA expression. (*p* = 0.9422, *<0.05, **<0.01, ***<0.001, ns, non-significant ≥ 0.05).

For differentially expressed BMPs/BMPRs (deBMPs/BMPRs), survival analysis was performed with prognostic information from the tumor samples of TCGA-LUAD (*n* = 513). Among eight deBMPs/BMPRs, only BMP5 (HR: 0.59, 95% CI: 0.44–0.79, *p*: 4e-04 < 0.001) and GDF10 (HR: 0.74, 95% CI: 0.56–0.99, *p*: 0.0444 < 0.05) had prognostic value, and the higher expression of both indicated a better prognosis in lung adenocarcinoma (**Figure 3A**). The results from the Kaplan–Meier plotter from 719 patients with lung adenocarcinoma also supported the prognostic value of BMP5 (HR: 0.48, 95% CI: 0.38–0.61, *p*: 9e-10 < 0.001) and GDF10 (HR: 0.73, 95% CI: 0.57–0.92, *p*: 0.0085 < 0.001) from TCGA-LUAD (**Figure 3B**). High expression of BMP2 (HR: 0.64, 95% CI: 0.51–0.81, *p*: 0.00016 < 0.001) suggested the short OS in the Kaplan–Meier plotter, but its expression (HR: 1.09, 95% CI: 0.82–1.46, *p*: 0.5583 > 0.05) did not have significant prognostic value in TCGA-LUAD. Compared with GDF10, the above results both suggested that BMP5 has high prognostic value and its increased expression has a high association with better prognosis in lung adenocarcinoma.

Multivariate survival analysis was performed *via* the Cox proportional hazard model. The results shown in the form of a forest plot revealed that BMP5 is an independent prognostic factor among the eight deBMPs/BMPRs (HR: 0.8416, 95% CI: 0.7738–0.9154, *p*: 5.75e-05 < 0.001). The lower the expression of BMP5, the shorter the OS, and the poorer the prognosis in lung adenocarcinoma (**Figure 3C**). Last, the correlation among eight deBMPs/BMPRs was analyzed in tumor tissues of TCGA-LUAD, BMP5, and GDF10, as prognostic factors were well correlated with other deBMPs/BMPRs (**Figure 3D**).

To further explore the value of BMP5 in clinical treatment, we first analyzed the correlation of mRNA expression between BMP5 and EGFR/STK11, which are critical molecular in targeted therapy and immunotherapy of lung adenocarcinoma. The results showed that there was a statistically positive correlation between BMP5 and EGFR expression (*p* = 0.013, *r* = 0.110) (**Figure 3E**), while BMP5 was not correlated with STK11 expression (*p* = 0.572, *r* = 0.024) (**Figure 3F**). In view of the critical role of EGFR and STK11 mutations in the efficacy of lung cancer treatment, we analyzed the differences of BMP5 mRNA expression in patients with and without EGFR and STK11 mutations. Results revealed that the expression level of BMP5 was significantly upregulated in patients with EGFR mutations (*p* = 0.0372) (**Figure 3G**), suggesting that BMP5 may be related to EGFR mutations and affect the treatment of EGFR-targeted therapy, but the specific mechanism remains unknown. Consistent with the correlation between BMP5 and STK11 expression, BMP5 expression was not correlated with STK11 mutations (*p* = 0.942) (**Figure 3H**).

### Association With Clinicopathological Features and Prognostic Value of the Risk Model

**Figure 4:**
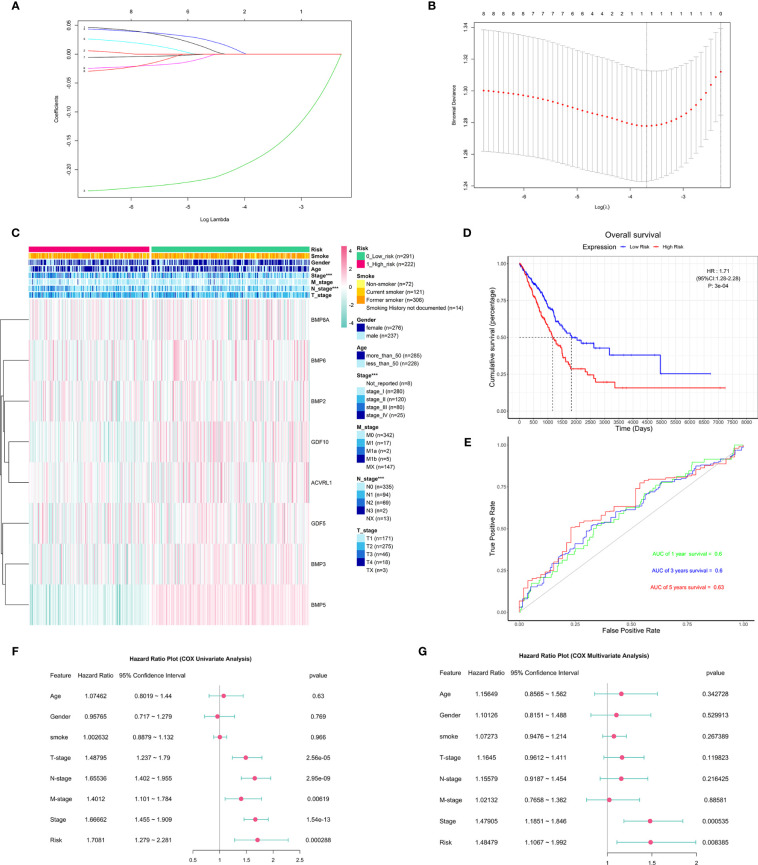
The construction and association of the risk model with clinicopathological features and prognostic value. **(A)** Distribution of least absolute shrinkage and selection operator (LASSO) coefficients for eight differentially expressed BMPs/BMPRs. **(B)** Partial likelihood deviation of the LASSO coefficient distribution. Vertical dashed lines indicate lambda.min (left) and lambda.1se (right). **(C)** Heatmap of the association between the risk model and clinicopathological features including age, gender, smoking, stage, T-stage, N-stage, and M-stage (***p < 0.001); smoking index information of smokers is not available. **(D)** The Kaplan–Meier curve of patients with low risk and high risk based on the risk model in TCGA-LUAD (HR: 1.71, 95% CI: 1.28–2.28, *p*: 3e-04). **(E)** The receiver operating characteristic (ROC) curve for the prognostic value of the risk model (AUC of 1-year survival: 0.6, 3-year survival: 0.6, 5-year survival: 0.63). **(F)** The forest plot of the univariate Cox proportional hazard model including clinicopathological features and risk. **(G)** The forest plot of the multivariate Cox proportional hazard model including clinicopathological features (stage: HR = 1.47905, 95% CI = 1.1851–1.846, *p* = 0.000535 < 0.001) and risk (HR: 1.48479, 95% CI: 1.1067–1.992, *p* = 0.008385 < 0.001).

To predict the clinical outcomes of lung adenocarcinoma using deBMPs/BMPRs, we applied the LASSO Cox regression algorithm to the eight genes in the TCGA-LUAD dataset. Only BMP5 was selected to build the risk model based on the minimum criteria, and its coefficient obtained from the LASSO algorithm was −0.1697154 for calculating the risk score for TCGA-LUAD (**Figures 4A, B**). Based on the median risk scores, the TCGA-LUAD dataset was divided into a high-risk group (*n* = 222) and a low-risk group (*n* = 291) to investigate the association with the clinicopathological features and prognostic roles of the risk model. Since treatment information was not available from the database for the 513 patients included, survival analysis was performed without taking into account the treatment differences.

To better understand the clinical outcomes of lung adenocarcinoma in low- and high-risk groups, we systematically analyzed the correlation between BMP5 in the risk model and the clinicopathologic features including age, gender, smoking, stage, T-stage, N-stage, and M-stage and found a relationship between the BMP5 risk model and stage (*p* < 0.001) and N-stage (*p* < 0.001) in lung adenocarcinoma (**Figure 4C**).

In investigating the prognostic value of the risk model, the Kaplan–Meier curve (**Figure 4D**) showed that high-risk scores indicated poor prognosis of lung adenocarcinoma, and the prognostic value of the risk model is obvious (HR: 1.71, 95% CI: 1.28–2.28, *p*: 3e-04); however, the receiver operating characteristic curve (ROC curve) found that the AUC was generally small (1-year survival: 0.6, 3-year survival: 0.6, 5-year survival: 0.63), and its diagnostic value was not satisfactory (**Figure 4E**).

Next, univariate and multivariate Cox regression analyses for the TCGA-LUAD were carried out to determine whether the risk signature was an independent prognostic indicator. Univariate Cox regression analysis found that risk score and TNM stage, including stage, T-stage, N-stage, and M-stage, were related to OS (**Figure 4F**). However, multivariate Cox regression analysis suggested that both stage and risk score were associated with prognosis in lung adenocarcinoma (**Figure 4G**).

### Correlation Between Modules and Phenotypes in WGCNA

**Figure 5:**
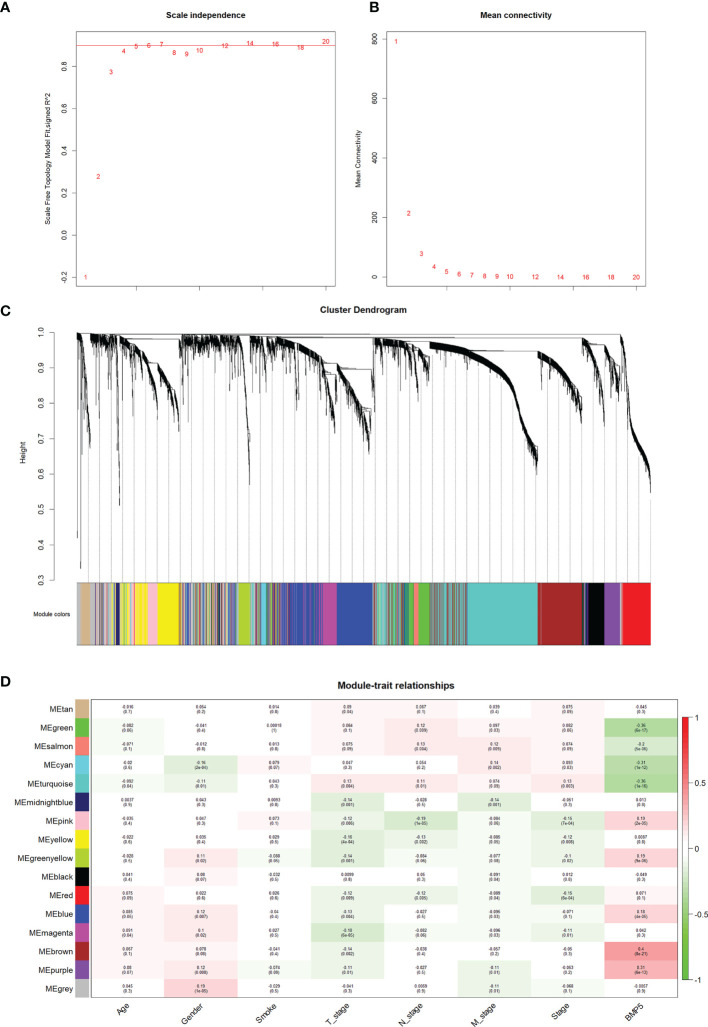
The identification of modules and their correlation with phenotypes particularly BMP5 expression in WGCNA. **(A, B)** The scale-free index and the average connectivity were both calculated under different *β* (the number in the figure indicates the corresponding soft threshold power). The approximate scale-free topology was achieved at a soft threshold power of 4. **(C)** Gene clustering tree diagram of 16 modules based on the common topological overlap, and each color module represents a module that contains a set of highly connected genes. **(D)** Correlation heatmap of different modules with various phenotypes including BMP5 expression. The numbers in brackets indicate the *p*-value, and the numbers without brackets indicate the correlation.

As described above, the current study calculated the MAD of each gene in 526 tumor tissues and 59 normal tissues of TCGA-LUAD, and the top 5,000 genes with the highest MAD values were selected for co-expression network construction. While moderately retaining the average connectivity of each gene node, we chose the appropriate weighting factor *β* to construct a scale-free network. Finally, the *β* value was determined to be 4 for co-expression network construction (**Figures 5A, B**), and a total of 16 modules were then identified (**Figure 5C**).

Based on the correlation between modules and clinicopathologic features, the modules significantly associated with BMP5 expression were selected (**Figure 5D**). The brown module was the most relevant one to BMP5 expression (coefficient: 0.4, *p*: 8e-21) and was chosen as the focus of subsequent research. The positive coefficient indicated a positive correlation between the screened module and BMP5, and a higher coefficient with a statistically significant *p*-value indicated a stronger correlation with BMP5 and prognosis in lung adenocarcinoma. As the above results show, the genes in the brown module may be regulated by BMP5 and play an important role in the prognosis of patients with lung adenocarcinoma.

### Function Enrichment of Module Genes and Identification of Hub Genes

**Figure 6:**
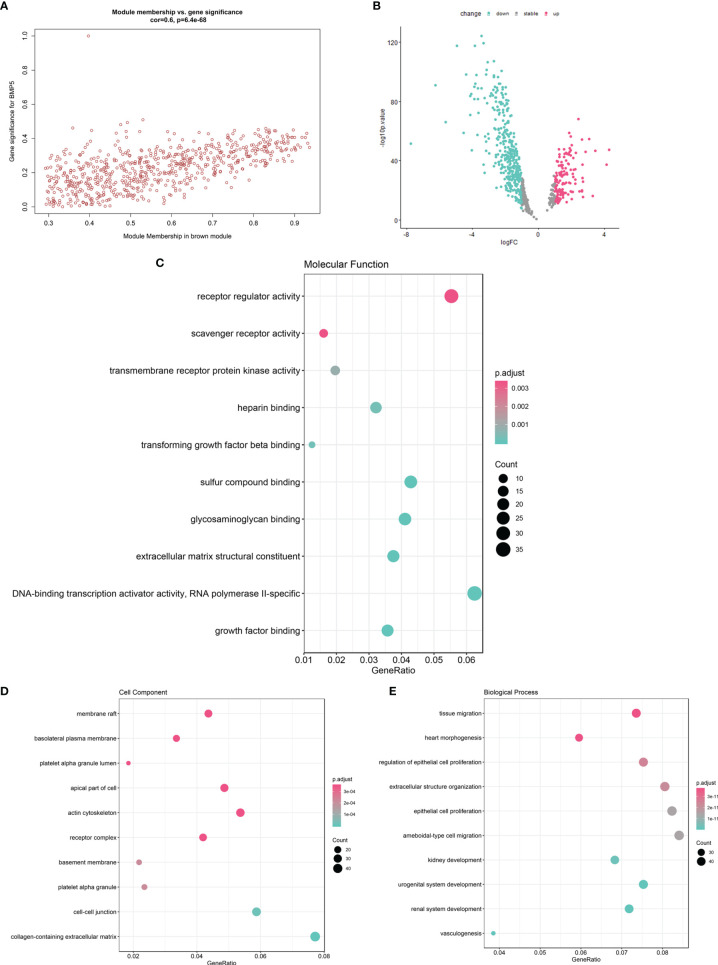
The function enrichment of brown module genes and the identification of hub genes with prognostic value. **(A)** The scatter diagram of correlation between brown module genes (*n* = 682) and BMP5 expression (correlation: 0.6, *p*-value: 6.4e-68 < 0.001). **(B)** The volcano plot of brown module gene expression profile in TCGA-LUAD indicating 537 DEGs (152 upregulated *vs.* 385 downregulated genes). **(C–E)** The GO function enrichment of 537 DEGs in terms of molecular function, cell component, and molecular function.

On the premise of considering the colinear relationship among module genes, we re-examined the correlation between the brown module and BMP5. A correlation coefficient of 0.6 with a *p*-value <0.001 fully confirmed the positive correlation between the module and BMP5 (**Figure 6A**). To further identify the prognostic factors regulated by BMP5, a total of 682 genes in the brown module were selected as module genes for further study (**Supplementary Table S2**). The differential expression analysis for module genes excluded 145 genes with stable expression (152 upregulated *vs.* 385 downregulated genes) in comparing tumor tissues with normal tissues in TCGA-LUAD (**Figure 6B** and **Supplementary Table S3**).

The 537 DEGs were analyzed for GO function enrichment. In molecular function terms of GO (**Figure 6C**), the DEGs were mainly enriched in “receptor regulator activity” (gene ratio > 0.04 and *p* < 0.001). In cell component terms (**Figure 6D**), the genes were mainly enriched in “actin cytoskeleton”, “apical part of cell”, “membrane raft”, and “receptor complex” (gene ratio > 0.04 and *p* < 0.0001). Although the molecular function “receptor regulator activity” is very common, the enriched DEGs under cell components such as “actin cytoskeleton” and “membrane raft” were associated with cell migration and tumor invasion. Interestingly, in terms of biological processes (**Figure 6E**), tissue migration was proven the most significant enrichment process. DEGs also were enriched in “ameboidal-type cell migration”, “epithelial cell proliferation”, “extracellular structure organization”, and “regulation of epithelial cell proliferation” (gene ratio > 0.06 and *p* < 1e-11). The above results of the function enrichment analysis suggested that the DEGs associated with BMP5 may play an important role in invasion and metastasis and affect prognosis in lung adenocarcinoma.

To screen prognostic genes from the above DEGs, univariate Cox was performed with *p <*0.01 used as the cutoff; 79 genes were selected as hub genes for subsequent analysis (**Supplementary Table S4**).

### Re-Establishment of the Risk Model

**Figure 7:**
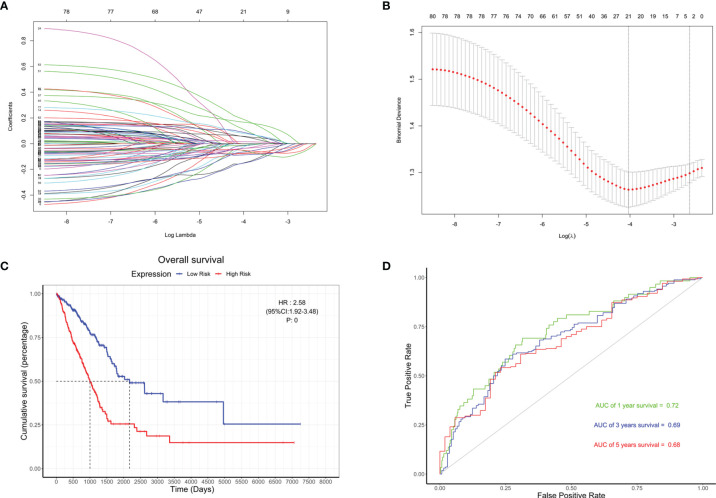
The re-establishment of the risk model and the evaluation of its prognostic value. **(A)** Distribution of least absolute shrinkage and selection operator (LASSO) coefficients for BMP5 and 79 hub genes. **(B)** Partial likelihood deviation of the LASSO coefficient distribution. Vertical dashed lines indicate lambda.min (left) and lambda.1se (right). **(C)** The Kaplan–Meier curve of patients with low risk and high risk based on the re-established risk model in TCGA-LUAD (HR: 2.58, 95% CI: 1.92–3.46, *p*: 0). **(D)** The receiver operating characteristic (ROC) curve for the prognostic value of the re-established risk model (AUC of 1-year survival: 0.72, 3-year survival: 0.69, 5-year survival: 0.68).

Based on the 79 hub genes and BMP5, LASSO analysis was performed to improve and re-establish the risk model (**Figures 7A, B**). A new risk model was constructed with BMP5 as the core gene and 20 others as hub genes (**Supplementary Table S5**). Compared with the risk model created before improvement, risk scores based on the new model showed better correlation with prognosis, and higher risk scores suggested poorer OS (HR: 2.58, 95% CI: 1.92–3.46, *p*: 0) in lung adenocarcinoma (**Figure 7C**). More importantly, the ROC curve showed that the new model improved the original prognostic accuracy of lung adenocarcinoma (AUC of 1-year survival: 0.72, 3-year survival: 0.69, and 5-year survival: 0.68), especially for 1-year survival (**Figure 7D**). Although BMP5 showed outstanding prognostic value that its low expression indicates poor prognosis, the evaluation of survival time is still not very ideal, which may be affected by individual differences and various treatment options to some extent.

### Prognostic Value of Hub Genes in the Risk Model

**Figure 8:**
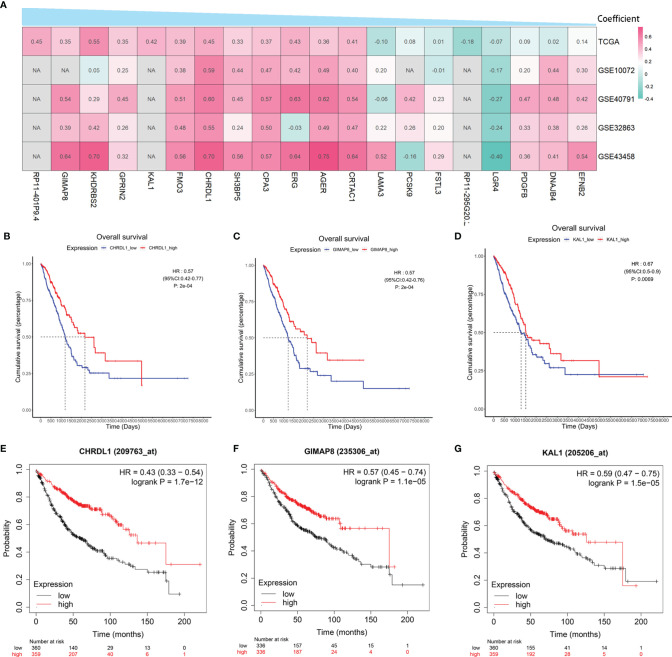
The correlation with BMP5 and prognostic value of hub genes in the re-established risk model. **(A)** Correlation heatmap of 20 hub genes with BMP5 in TCGA-LUAD and four GEO datasets. **(B–D)** The Kaplan–Meier curves of CHRDL1, GIMAP8, and KAL1 in the tumor samples of TCGA-LUAD (*n* = 513). CHRDL1 (HR: 0.57, 95% CI: 0.42–0.77, *p*: 2e-04 < 0.001), GIMAP8 (HR: 0.57, 95% CI: 0.42–0.76, *p*: 2e-04 < 0.001), and KAL1 (HR: 0.67, 95% CI: 0.50–0.90, *p*: 0.0069 < 0.001). **(E–G)** The Kaplan–Meier curves of CHRDL1, GIMAP8, and KAL1 in lung adenocarcinoma of Kaplan–Meier plotter (*n* = 719). CHRDL1 (HR: 0.43, 95% CI: 0.33–0.54, *p*: 1.7e-12 < 0.001), GIMAP8 (HR: 0.57, 95% CI: 0.45–0.74, *p*: 1.1e-05 < 0.001), and KAL1 (HR: 0.59, 95% CI: 0.47–0.75, *p*: 1.5e-05 < 0.001). NA, not available.

To verify the correlation between the 20 hub genes in the risk model and BMP5, the above four GEO datasets were used as validation datasets and used for correlation analysis. A heatmap was drawn according to the coefficients in the risk model from the largest to the smallest, and it was found that the correlation between hub genes and BMP5 was essentially consistent among TCGA-LUAD and the four verification sets (**Figure 8A**). Besides BMP5, the prognostic value of each hub gene in the risk model was evaluated, and Kaplan–Meier curves show that almost all hub genes had a high prognostic value, especially CHRDL1, GIMAP8, and KAL1 (**Figures 8B**–**D**). This result was supported by the results of the Kaplan–Meier plotter (**Figures 8E**–**G**). The high expression of the three genes indicated a better prognosis consistent with BMP5, suggesting that CHRDL1, GIMAP8, and KAL1 may be the key molecules for prognosis influenced by BMP5 in lung adenocarcinoma.

### Clinical Characteristics of Patients With Lung Adenocarcinoma in TMA

As shown in **Table 4**, among 93 patients with lung adenocarcinoma, 44 (47.3%) patients were men and 49 (52.7%) patients were women. The median age of the patients was 57 years (range 21–74) and 37 (39.8%) patients had a history of smoking. Except for nine cases (9.7%) with well-differentiated or moderately well-differentiated tumors, the rest (90.3%) were diagnosed with poorly differentiated, moderately differentiated, or poorly moderately differentiated tumors. According to the eighth edition of UICC/AJCC lung cancer stage classification (2017), 33 patients (35.5%) were classified as stage I–II and the rest (64.5%) were classified as stage III. Treatment information was not available for the 93 patients included.

**Table 4:** Clinical characteristics of 93 patients with lung adenocarcinoma.

Characteristics	No. of patients (%)
Age (years) (median, range)	57 (21, 74)
≤57	40 (43.0%)
>57	53 (57.0%)
Gender	
Male	44 (47.3%)
Female	49 (52.7%)
Smoking	
No	56 (60.2%)
Yes	37 (39.8%)
Tumor differentiation grade	
Poor	26 (28.0%)
Poor–moderate	30 (32.3%)
Moderate	28 (30.0%)
Well/moderate–well	9 (9.7%)
Pathological tumor (T) status^a^	
T1–T2	62 (66.7%)
T3–T4	31 (33.3%)
Pathological node (N) status^a^	
N0–N1	34 (36.6%)
N2–N3	59 (63.4%)
Clinical stage^a^	
I–II	33 (35.5%)
III	60 (64.5%)

a Pathological tumor (T) status, pathological node (N) status, and clinical stage are from the eighth edition of UICC/AJCC lung cancer stage classification (2017).

### Verification of BMP5 Expression and Assessment of Its Association With Prognosis in TMA by IHC Staining

**Figure 9:**
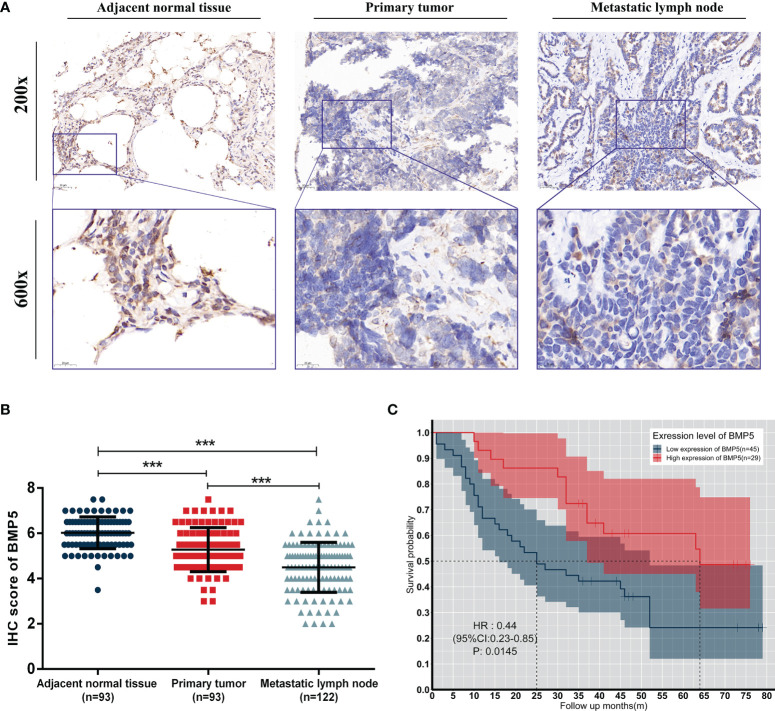
Expression levels and prognosis values of BMP5 protein in patients with lung adenocarcinoma. **(A)** Representative IHC staining of BMP5 in sections from adjacent normal tissues, primary tumors, and metastatic lymph nodes. **(B)** The IHC score of METTL3 in adjacent normal tissues was higher than that of primary tumors and metastatic lymph nodes. Statistical significance was determined by a two-tailed, paired Student’s *t*-test. **(C)** Kaplan–Meier analysis of overall survival according to high and low BMP5 protein expression in 93 lung cancer patients (***p < 0.001).

The average IHC score of BMP5 protein expression in primary tumor (*n* = 93) was 5.27, which was lower than the score in adjacent normal tissues (*n* = 93, average IHC score = 6.02). Metastatic lymph nodes (*n* = 122) showed even lower BMP5 expression (average IHC score = 4.49) than primary tumor. Overall, BMP5 expression was higher in adjacent normal tissues than primary tumor tissues and was the lowest in metastatic lymph nodes (**Figures 9A, B**).

A total of 74 patients with complete follow-up data were included and divided into two groups (high expression of BMP5, *n* = 29; low expression of BMP5, *n* = 45) based on mean IHC score of BMP5 as the cutoff value in their primary tumors. Kaplan–Meier survival curve revealed that BMP5 expression levels were significantly associated with overall survival in lung adenocarcinoma patients. The overall survival was longer in patients with high BMP5 expression compared with patients with low expression (HR: 0.44, 95% CI: 0.23–0.85, *p* = 0.0145) (**Figure 9C**).

## Discussion

BMPs are multifunctional cytokines that fulfill their biological function through activation of canonical SMAD-dependent signaling pathway binding to type I and II BMPRs. At present, approximately 20 BMPs have been identified; however, most studies involving BMPs in lung cancer have focused on BMP2, BMP4, and BMP7. Magdalena Bieniasz et al. found a positive correlation between VEGF and BMP2, underlining the importance of BMP2 in angiogenesis in lung cancer (28). As a close relative of BMP2, the upregulation of BMP4 has been proven to be strongly associated with EGFR-TKI resistance and fatty acid metabolism in lung cancer (29). Additionally, BMP7 was found to inhibit progression of small cell lung cancer by inducing cell cycle arrest (30). Unfortunately, the screening of BMPs in the above studies was either from results of extrapulmonary tumor studies or from small-scale tests in a small sample of a population with lung cancer, without considering the effects of histology type. At present, differential expression analysis of BMPs/BMPRs in a large sample is lacking, and the prognostic value of BMPs/BMPRs is far from sufficient in lung cancer, especially for lung adenocarcinoma.

In systematically screening BMPs, the current study identified eight deBMPs/BMPR in TCGA-LUAD by comparing 526 tumor tissues with 347 normal tissues. The subsequent validation in four GEO datasets screened out five stably downregulated BMPs/BMPRs (BMP2, BMP5, BMP6, BMP3B/GDF10, and ACVRL1). To evaluate the prognostic value of these five deBMPs/BMPRs, a series of survival analyses including Kaplan–Meier curve and univariate and multivariate Cox regression analyses were carried out, and BMP5 was identified as an independent protective prognostic factor in lung adenocarcinoma. We found that BMP5 expression was significantly correlated with EGFR expression and mutations, suggesting that BMP5 may play a role in targeted therapy. Based on BMP5 and closely related hub genes such as CHRDL1, GIMAP8, and KAL1, we constructed and improved a prognostic risk model that effectively evaluated the prognosis of lung adenocarcinoma. Subsequently, we verified the protein expression of BMP5 in TMA by IHC staining. The results showed that BMP5 expression was low in primary lung adenocarcinoma compared with adjacent normal tissues, and was even lower in metastatic lymph nodes. Consistent with public database analysis, higher expression of BMP5 protein in lung adenocarcinoma indicated better prognosis.

BMP5, as a tumor suppressor, has been previously studied in myeloma, adrenocortical carcinoma, breast cancer, and colorectal cancer (31). Regarding the function of BMP5, Mathilde Romagnoli et al. reported that repression of BMP5 induced epithelial-to-mesenchymal transition and promoted the metastasis of breast cancer (32). Although a study based on 76 lung cancer samples initially found that BMP5 was downregulated, the prognostic value and potential function of BMP5 in lung adenocarcinoma are still unclear (33). After confirmation of the prognostic value of BMP5 in our study, it is reasonable to speculate that BMP5 may influence prognosis in synergy with CHRDL1, GIMAP8, and KAL1 according to the results of the weighted gene correlation network.

Developmental studies confirmed that BMP signaling could be suppressed by cysteine-rich domain proteins that sequester ligands from the BMPRs such as chordin (34). Chordin-like 1(CHRDL1), as a secreted antagonist of BMP signaling, has been previously reported to predominantly suppress BMP4-induced migration and invasion, and its higher expression indicates better clinical outcomes in breast cancer (35). Interestingly, CHRDL1, the BMP antagonist, was found to be co-expressed with BMP5, and both were screened for their significant prognostic protective value in lung adenocarcinoma in the current study. It is necessary for CHRDL1 to explore the regulatory relationship of CHRDL1 with BMP5 and its role in lung adenocarcinoma.

Currently, immunotherapy based on immune checkpoint PD-L1 (programmed death ligand 1) has made remarkable progress in the treatment of advanced lung cancer, accompanying the problem of limited beneficiaries (36). The GIMAP (GTPase of immunity-associated proteins) has been implicated in the regulation of immune cell survival (37). As early as in 2008, a study reported that GIMAP8 was significantly reduced in the lung tumor tissues and suggested a potential role in tumor immunity (38). Consistently, we also found that GIMAP8, as a BMP5 co-expressed hub gene, was downregulated in tumor tissues, and its higher expression indicated better prognosis in lung adenocarcinoma. The application of GIMAP8 or even BMP5 in immunotherapy will be the focus of our next study.

The unified nomenclature for Kallmann syndrome 1 gene (KAL1) and anosmin-1 is ANOS1 (39). At present, the functions of ANOS1 in tumors are mainly concentrated in gastrointestinal tumors. A series of studies in gastric cancer by Mitsuro Kanda et al. found that prognosis was worse for patients with preoperative serum ANOS1 ≥600 pg/ml compared with those with <600 pg/ml (40), and serum levels of ANOS1 have been suggested as a diagnostic biomarker based on a prospective multicenter observational study (41). However, the diagnostic value of ANOS1 and its regulatory relationship with BMP5 are currently unclear, and there is a large value in future exploration in lung adenocarcinoma.

Compared with other studies focused on BMPs/BMPRs in lung cancer, the present research is a systematic screening study of BMPs/BMPRs based on large-sample RNA-seq data. On the premise of fully considering the influence of histology types in this study, we focused on lung adenocarcinoma to explore the expression differences and prognostic correlation of BMPs/BMPRs. Finally, we identified BMP5 as having significantly differential expression and superior prognostic value, rather than BMP2, BMP4, and BMP7, which have been extensively explored in lung cancer. To some extent, the current results provide a direction for the subsequent studies on the mechanism of BMPs in lung adenocarcinoma. In addition, the WGCNA identified hub genes co-expressed with BMP5 and, for the subsequent, puts forward an important series of thoughts. First, CHRDL1 as a BMP signaling antagonist suppresses tumor metastasis but co-expressed with BMP5 in lung adenocarcinoma. Therefore, exploration of the regulatory mechanisms of both will further the understanding of lung cancer metastasis. Second, GIMAP8 is a potential immunotherapy target and will be the focus of subsequent studies to improve the efficacy of immunotherapy on the basis of fully understanding the relationship between GIMAP8 and BMP5. Third, the diagnostic value of KAL1 as a mature diagnostic biomarker in gastric cancer is also worthy looking forward to in lung adenocarcinoma.

The current research completed a systematic screening of BMPs and preliminary functional exploration in lung adenocarcinoma; however, the biggest deficiency is that the above results are mainly based on bioinformatics analysis of public databases. Although the sample size included is large and the results have been repeatedly verified by different cohorts, these conclusions still lack necessary experimental evidence. Subsequent studies will conduct experiments on the detailed mechanisms of BMP5 in lung adenocarcinoma, especially on the regulatory relationship between BMP5 and the three hub genes. Based on significant differential expression and superior prognostic value, BMP5 has the potential to become a crucial target for the treatment of lung adenocarcinoma.

## Conclusion

We screened and verified four differentially expressed BMPs (BMP2, BMP5, BMP6, and GDF10) and one BMPR (ACVRL1), which all were downregulated in lung adenocarcinoma tissues. BMP5 was identified as an independent protective prognostic factor, and higher BMP5 expression indicated better clinical outcomes in lung adenocarcinoma. The correlation between BMP5 and EGFR expression and mutations suggests that BMP5 may affect EGFR-targeted therapy in lung adenocarcinoma. Based on the co-expression network of BMP5, 79 hub genes were selected, and their functions were enriched in cell migration and tumor invasion. The risk model was established around BMP5 and its hub genes and constantly improved. Subsequently, the risk model combining 20 hub genes including CHRDL1, GIMAP8, KAL1, and BMP5 as the core showed significant prognostic correlation and excellent prognostic value. Finally, IHC staining in TMA revealed that the expression level and prognostic value of BMP5 protein were consistent with public database analysis. In conclusion, BMP5 is differentially expressed and can accurately evaluate prognosis as an independent protective factor in lung adenocarcinoma. BMP5 is expected to become an important target for the treatment of lung adenocarcinoma. Future research will focus on the detailed mechanisms of BMP5, especially the regulatory relationship with the three hub genes.

## Data Availability Statement

The datasets presented in this study can be found in online repositories. The names of the repository/repositories and accession number(s) can be found in the article/**Supplementary Material**.

## Ethics Statement

The studies involving human participants were reviewed and approved by the Institutional Ethics Committee of Tongji Medical College, Huazhong University of Science and Technology. The patients/participants provided their written informed consent to participate in this study.

## Author Contributions

HX: ideas incubation, study design, workflow construction, data analysis, data interpretation, writing—original draft, and writing—review and editing. YL: ideas incubation, study design, and writing—review and editing. WM: study design, data collection, data analysis, writing—original draft, and writing—review and editing. RZ: literature search, data collection, writing—original draft, and writing—review and editing. DL: study design, workflow construction, figures, data interpretation, and writing—review and editing. KL: literature search, data collection, and writing—original draft. YM: data collection and writing—review and editing. JC: data collection and writing—review and editing. YW: data collection and writing—review and editing. All authors contributed to the article and approved the submitted version.

## Conflict of Interest

The authors declare that the research was conducted in the absence of any commercial or financial relationships that could be construed as a potential conflict of interest.

## Publisher’s Note

All claims expressed in this article are solely those of the authors and do not necessarily represent those of their affiliated organizations, or those of the publisher, the editors and the reviewers. Any product that may be evaluated in this article, or claim that may be made by its manufacturer, is not guaranteed or endorsed by the publisher.

## Supplementary Material

Supplementary Figure S1 The distribution characteristics of 11 BMPs and 7 BMPRs in 4 GEO verification datasets. **(A)** Box plot indicating the expression distribution of 10 BMPs (lack of GDF7) and 7 BMPRs between tumor and normal tissues in GSE10072; **(B)** Box plot indicating the expression distribution of 10 BMPs (lack of GDF7) and 7 BMPRs between tumor and normal tissues in GSE40791; **(C)** Box plot indicating the expression distribution of 11 BMPs and 7 BMPRs between tumor and normal tissues in GSE32863; **(D)** Box plot indicating the expression distribution of 11 BMPs and 7 BMPRs between tumor and normal tissues in GSE43458.

Supplementary Figure S2 The Kaplan Meier curves of the other differentially expressed BMPs/BMPRs in TCGA-LUAD. **(A)** Kaplan Meier curve of ACVRL1 (HR: 0.94, 95% CI: 0.7~1.25, p: 0.6605 > 0.05); **(B)** Kaplan Meier curve of BMP3 (HR: 0.81, 95% CI: 0.61~1.08, p: 0.1576 > 0.05); **(C)** Kaplan Meier curve of BMP6 (HR: 0.95, 95% CI: 0.71~1.26, p: 0.7105 > 0.05); **(D)** Kaplan Meier curve of BMP8A (HR: 1.2, 95% CI: 0.9~1.61, p: 0.2098 > 0.05); **(E)** Kaplan Meier curve of GDF5 (HR: 0.84, 95% CI: 0.63~1.12, p: 0.2266 > 0.05).

Supplementary Figure S3 The Kaplan Meier curves of the other differentially expressed BMPs/BMPRs in lung adenocarcinoma from the Kaplan Meier plotter. **(A)** Kaplan Meier curve of ACVRL1 (HR: 0.91, 95%CI: 0.71-1.16, p:0.44>0.05); **(B)** Kaplan Meier curve of BMP3 (HR: 0.82, 95%CI: 0.65-1.04, p:0.095>0.05); **(C)** Kaplan Meier curve of BMP6 (HR: 0.92, 95%CI: 0.73-1.16, p:0.49>0.05); **(D)** Kaplan Meier curve of BMP8A (HR: 1.09, 95%CI: 0.86-1.37, p:0.48>0.05); **(E)** Kaplan Meier curve of BMP14/GDF5 (HR: 1.01, 95%CI: 0.8-1.28, p:0.9>0.05).

Supplementary Figure S4 The Kaplan Meier curves of the other hub genes with significant prognostic value (p < 0.001) in TCGA-LUAD. **(A)** Kaplan Meier curve of AGER (HR: 0.67, 95% CI: 0.5~0.9, p: 0.0071 < 0.001); **(B)** Kaplan Meier curve of CRTAC1 (HR: 0.67, 95% CI: 0.5~0.9, p: 0.0081 < 0.001); **(C)** Kaplan Meier curve of DNAJB4 (HR: 1.52, 95% CI: 1.14~2.04, p:0.0049 < 0.001); **(D)** Kaplan Meier curve of KHDRBS2 (HR: 0.65, 95% CI: 0.48~0.87, p: 0.0036 < 0.001); **(E)** Kaplan Meier curve of LGR4 (HR: 1.56, 95% CI: 1.17~2.09, p: 0.0027 < 0.001); **(F)** Kaplan Meier curve of PDGFB (HR: 1.64, 95% CI: 1.22~2.19, p: 0.001).

Supplementary Figure S5 The Kaplan Meier curves of the other hub genes in lung adenocarcinoma from the Kaplan Meier plotter. **(A)** Kaplan Meier curve of AGER (HR: 0.8, 95% CI: 0.63~1.01, p: 0.058 > 0.05); **(B)** Kaplan Meier curve of CRTAC1 (HR: 0.8, 95% CI: 0.63~1.02, p: 0.074 > 0.05); **(C)** Kaplan Meier curve of DNAJB4 (HR: 0.62, 95% CI: 0.49~0.78, p: 5.5e-05 < 0.001); **(D)** Kaplan Meier curve of KHDRBS2 (HR: 1.04, 95% CI: 0.82~1.31, p: 0.74 > 0.05); **(E)** Kaplan Meier curve of LGR4 (HR:0.61, 95% CI: 0.48~0.77, p: 3.5e-05 < 0.001); **(F)** Kaplan Meier curve of PDGFB/SSV (HR: 1.4, 95% CI: 1.11~1.77, p: 0.0042 < 0.001).

Supplementary Table S1 The expression matrix of 11 BMPs and 7 BMPRs in the dataset combining TCGA-LUAD and GTEx-LUNG.

Supplementary Table S2 The genes (n = 682) in the brown module of WGCNA.

Supplementary Table S3 The differentially expressed genes among brown module genes in TCGA-LUAD.

Supplementary Table S4 The univariate Cox proportional hazard model of hub genes as potential prognostic factors in module genes.

Supplementary Table S5 The re-established risk model based on BMP5 and 20 hub genes.
