# Supplementary figures and images for: The Prognostic Value of Bone Morphogenetic Proteins and Their Receptors in Lung Adenocarcinoma
Source: Front Oncol. 2021 Oct 22;11:608239. doi: 10.3389/fonc.2021.608239 (PMC8569625; doi:10.3389/fonc.2021.608239)

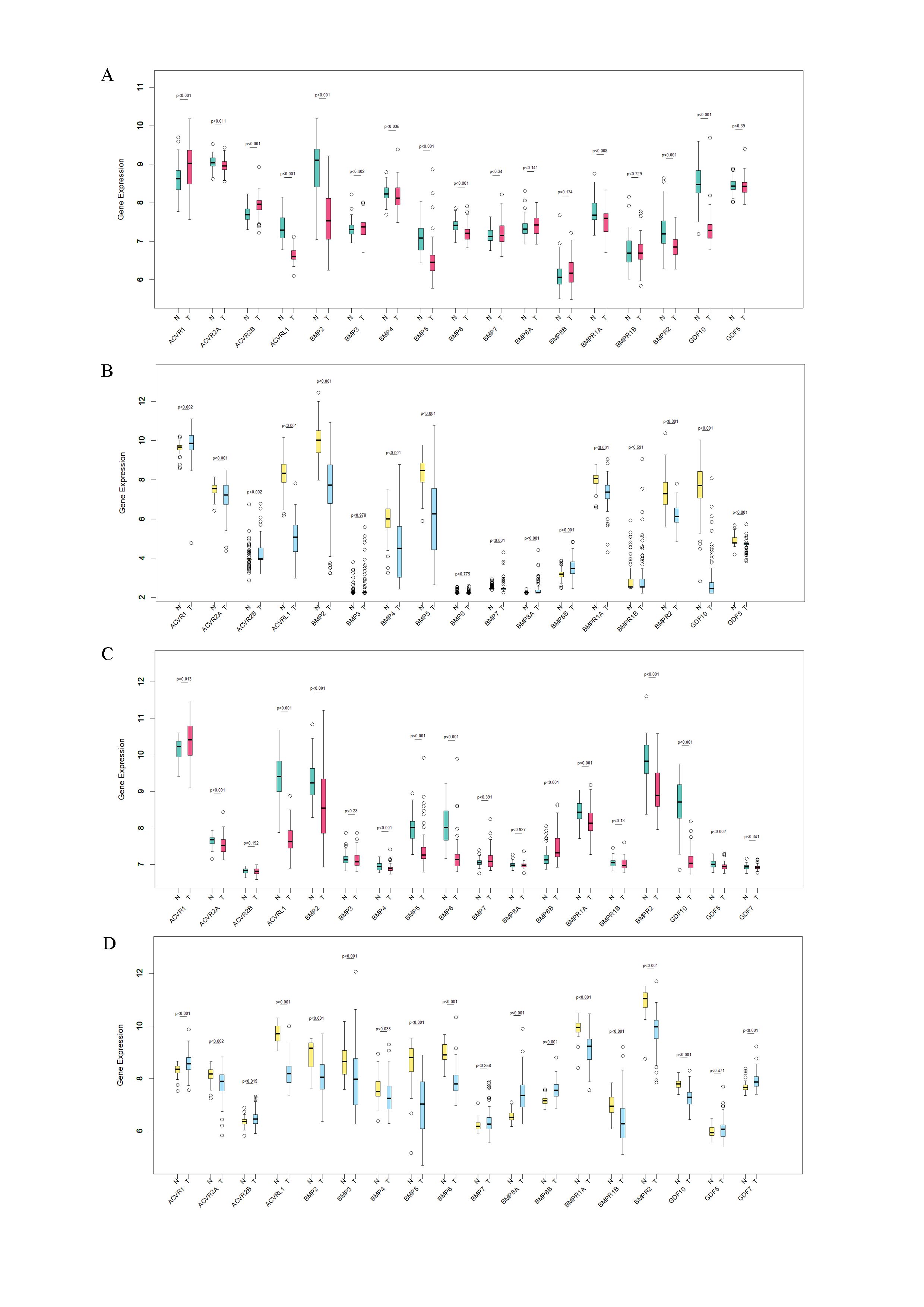

Supplement: Supplementary Figure S1 — The distribution characteristics of 11 BMPs and 7 BMPRs in 4 GEO verification datasets. (A) Box plot indicating the expression distribution of 10 BMPs (lack of GDF7) and 7 BMPRs between tumor and normal tissues in GSE10072; (B) Box plot indicating the expression distribution of 10 BMPs (lack of GDF7) and 7 BMPRs between tumor and normal tissues in GSE40791; (C) Box plot indicating the expression distribution of 11 BMPs and 7 BMPRs between tumor and normal tissues in GSE32863; (D) Box plot indicating the expression distribution of 11 BMPs and 7 BMPRs between tumor and normal tissues in GSE43458. [file Image_1.tif]

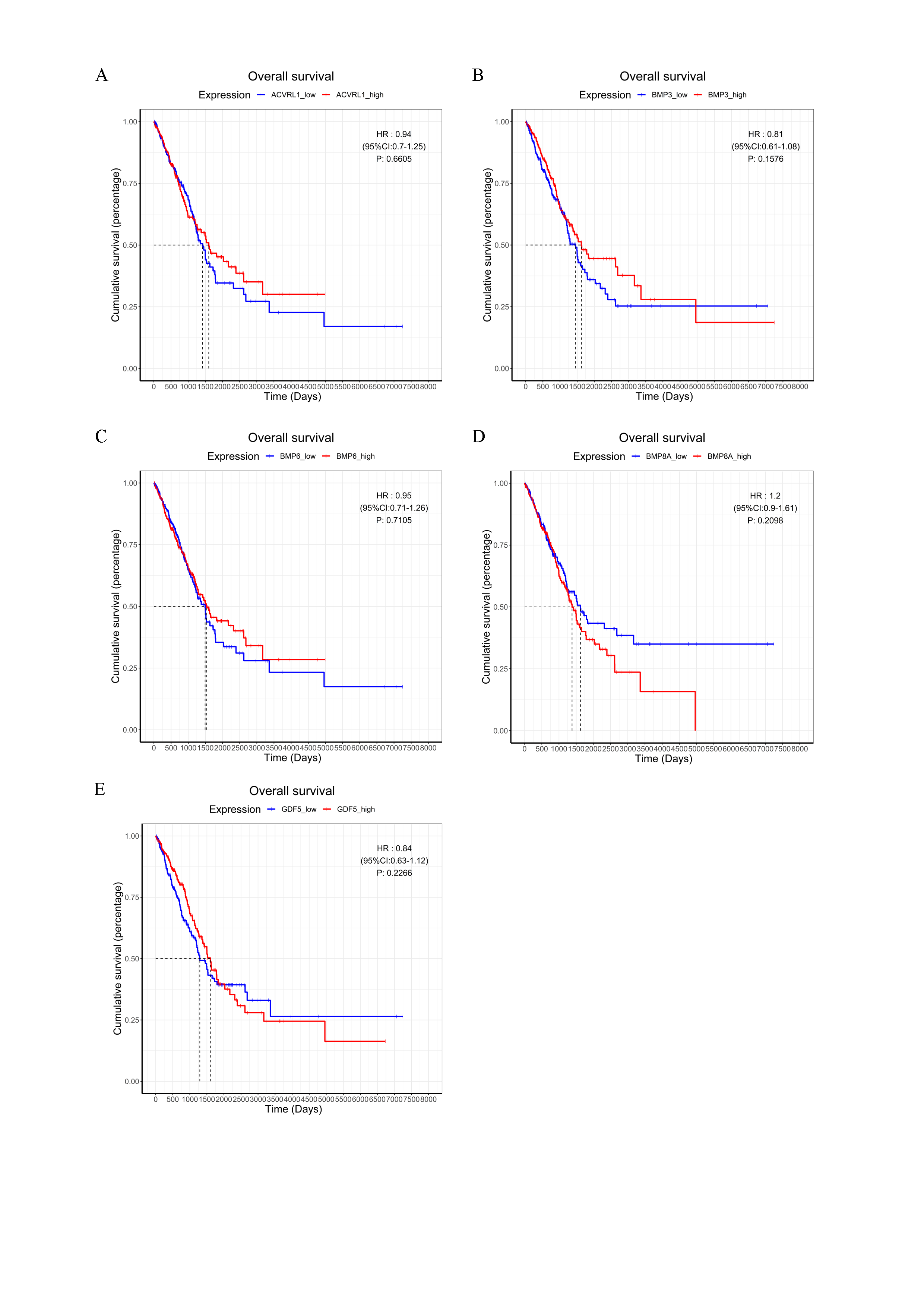

Supplement: Supplementary Figure S2 — The Kaplan Meier curves of the other differentially expressed BMPs/BMPRs in TCGA-LUAD. (A) Kaplan Meier curve of ACVRL1 (HR: 0.94, 95% CI: 0.7~1.25, p: 0.6605 > 0.05); (B) Kaplan Meier curve of BMP3 (HR: 0.81, 95% CI: 0.61~1.08, p: 0.1576 > 0.05); (C) Kaplan Meier curve of BMP6 (HR: 0.95, 95% CI: 0.71~1.26, p: 0.7105 > 0.05); (D) Kaplan Meier curve of BMP8A (HR: 1.2, 95% CI: 0.9~1.61, p: 0.2098 > 0.05); (E) Kaplan Meier curve of GDF5 (HR: 0.84, 95% CI: 0.63~1.12, p: 0.2266 > 0.05). [file Image_2.tif]

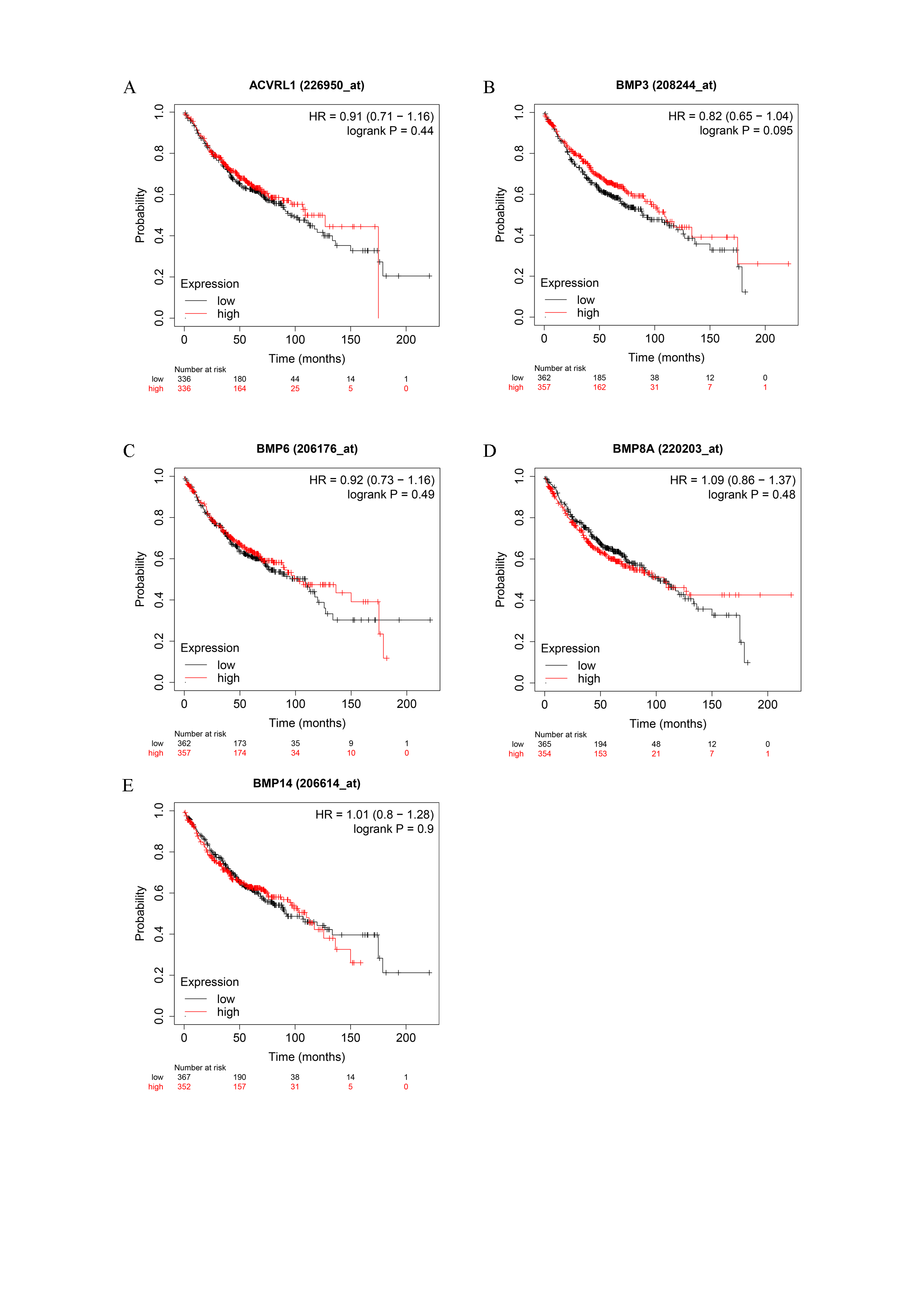

Supplement: Supplementary Figure S3 — The Kaplan Meier curves of the other differentially expressed BMPs/BMPRs in lung adenocarcinoma from the Kaplan Meier plotter. (A) Kaplan Meier curve of ACVRL1 (HR: 0.91, 95%CI: 0.71-1.16, p:0.44>0.05); (B) Kaplan Meier curve of BMP3 (HR: 0.82, 95%CI: 0.65-1.04, p:0.095>0.05); (C) Kaplan Meier curve of BMP6 (HR: 0.92, 95%CI: 0.73-1.16, p:0.49>0.05); (D) Kaplan Meier curve of BMP8A (HR: 1.09, 95%CI: 0.86-1.37, p:0.48>0.05); (E) Kaplan Meier curve of BMP14/GDF5 (HR: 1.01, 95%CI: 0.8-1.28, p:0.9>0.05). [file Image_3.tif]

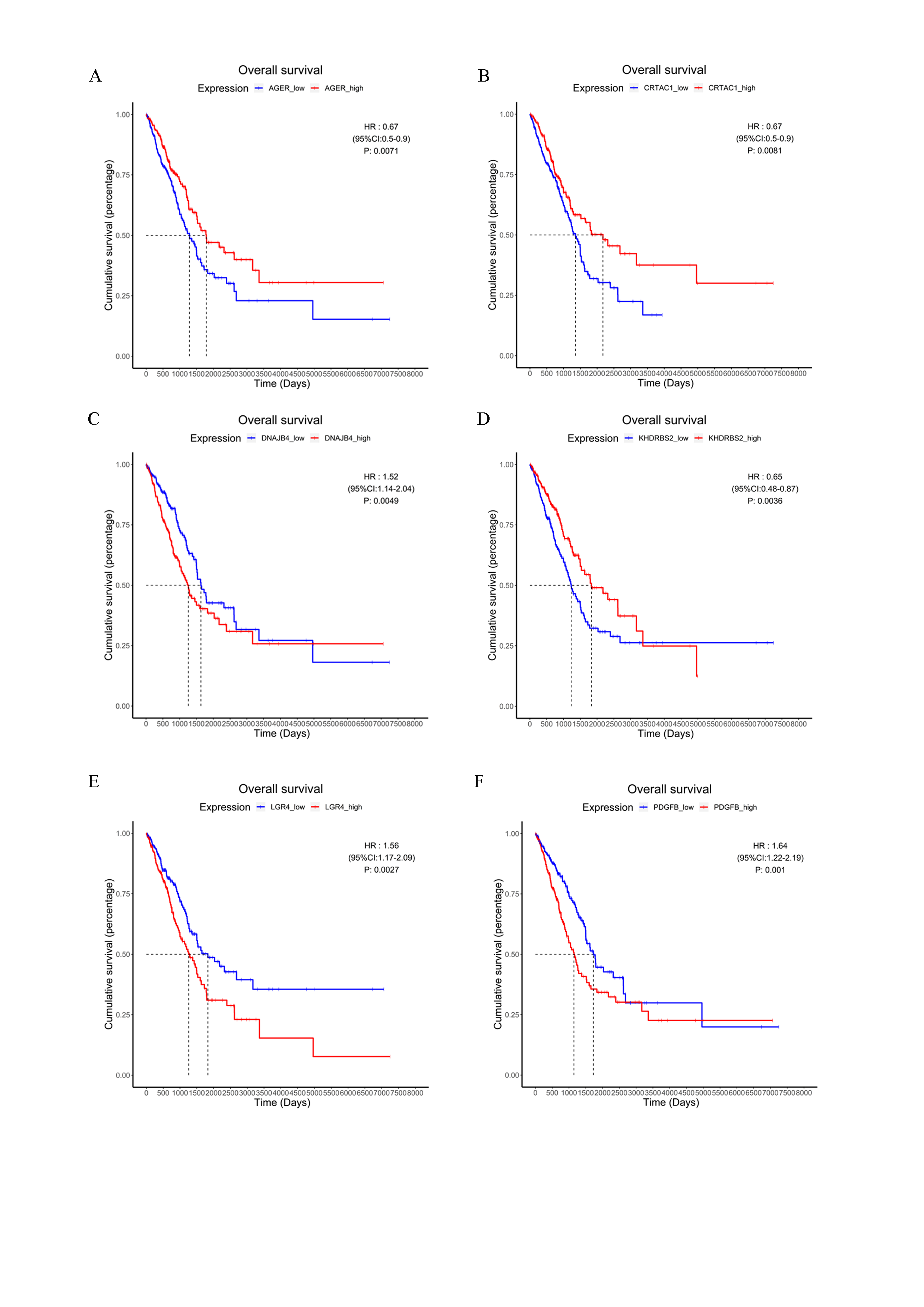

Supplement: Supplementary Figure S4 — The Kaplan Meier curves of the other hub genes with significant prognostic value (p < 0.001) in TCGA-LUAD. (A) Kaplan Meier curve of AGER (HR: 0.67, 95% CI: 0.5~0.9, p: 0.0071 < 0.001); (B) Kaplan Meier curve of CRTAC1 (HR: 0.67, 95% CI: 0.5~0.9, p: 0.0081 < 0.001); (C) Kaplan Meier curve of DNAJB4 (HR: 1.52, 95% CI: 1.14~2.04, p:0.0049 < 0.001); (D) Kaplan Meier curve of KHDRBS2 (HR: 0.65, 95% CI: 0.48~0.87, p: 0.0036 < 0.001); (E) Kaplan Meier curve of LGR4 (HR: 1.56, 95% CI: 1.17~2.09, p: 0.0027 < 0.001); (F) Kaplan Meier curve of PDGFB (HR: 1.64, 95% CI: 1.22~2.19, p: 0.001). [file Image_4.tif]

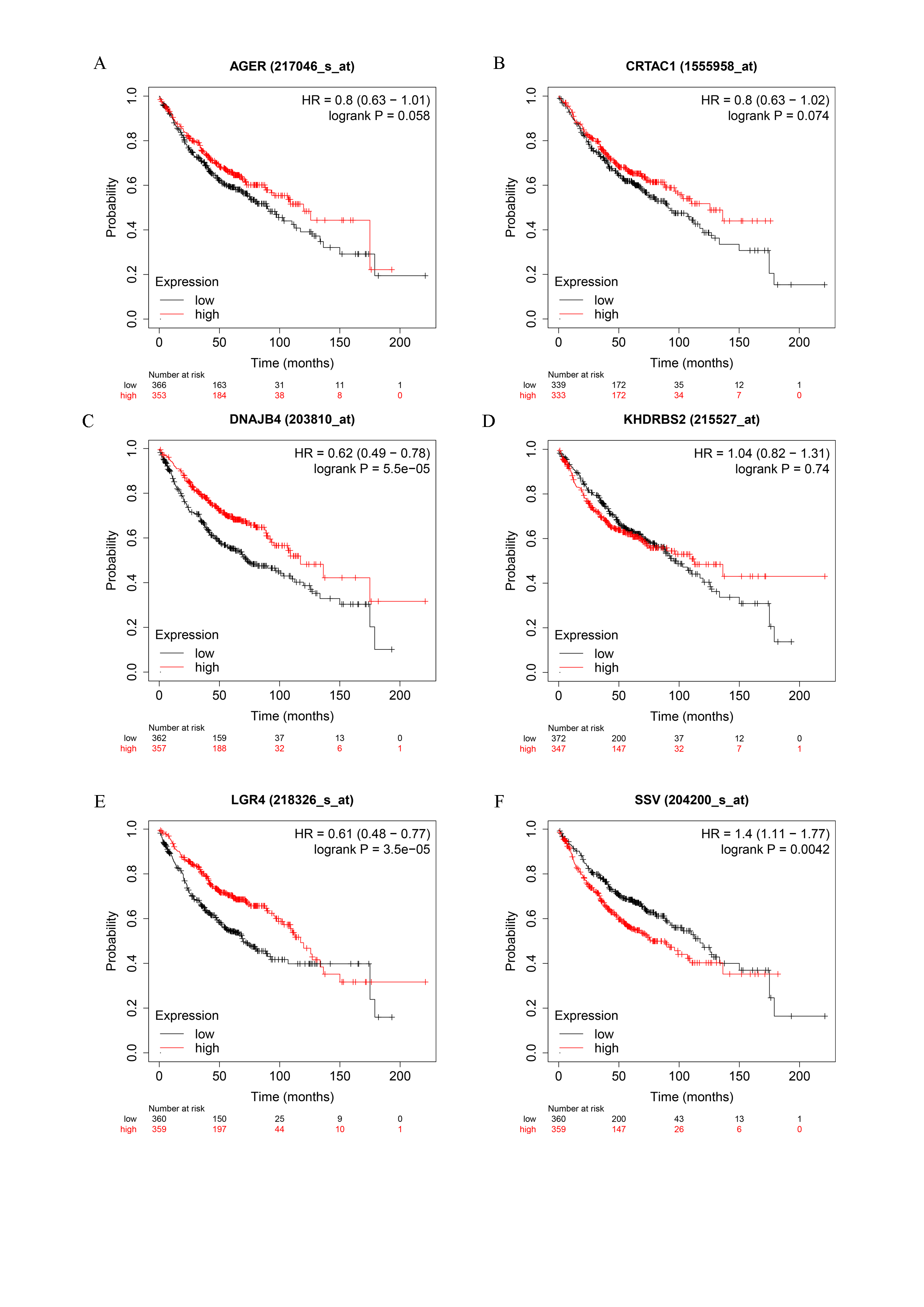

Supplement: Supplementary Figure S5 — The Kaplan Meier curves of the other hub genes in lung adenocarcinoma from the Kaplan Meier plotter. (A) Kaplan Meier curve of AGER (HR: 0.8, 95% CI: 0.63~1.01, p: 0.058 > 0.05); (B) Kaplan Meier curve of CRTAC1 (HR: 0.8, 95% CI: 0.63~1.02, p: 0.074 > 0.05); (C) Kaplan Meier curve of DNAJB4 (HR: 0.62, 95% CI: 0.49~0.78, p: 5.5e-05 < 0.001); (D) Kaplan Meier curve of KHDRBS2 (HR: 1.04, 95% CI: 0.82~1.31, p: 0.74 > 0.05); (E) Kaplan Meier curve of LGR4 (HR:0.61, 95% CI: 0.48~0.77, p: 3.5e-05 < 0.001); (F) Kaplan Meier curve of PDGFB/SSV (HR: 1.4, 95% CI: 1.11~1.77, p: 0.0042 < 0.001). [file Image_5.tif]
